# Which Factors Influence Healthy Aging? A Lesson from the Longevity Village of Bama in China

**DOI:** 10.14336/AD.2022.1108

**Published:** 2023-06-01

**Authors:** Wei Zhang, Qingyun Huang, Yongxin Kang, Hao Li, Guohe Tan

**Affiliations:** ^1^Department of Human Anatomy, Institute of Neuroscience and Guangxi Key Laboratory of Brain Science, Guangxi Health Commission Key Laboratory of Basic Research on Brain Function and Disease, School of Basic Medical Sciences, Guangxi Medical University, Nanning, Guangxi, China.; ^2^Key Laboratory of Longevity and Aging-related Diseases of Chinese Ministry of Education, Nanning, Guangxi, China.; ^3^Collaborative Innovation Centre of Regenerative Medicine and Medical BioResource Development and Application Co-constructed by the Province and Ministry, Guangxi Key Laboratory of Regenerative Medicine, Nanning, Guangxi, China.; ^4^China-ASEAN Research Center for Innovation and Development in Brain Science, Nanning, Guangxi, China.

**Keywords:** longevity, bama, healthy aging, gene polymorphism, environment

## Abstract

A growing aging population is associated with increasing incidences of aging-related diseases and socioeconomic burdens. Hence, research into healthy longevity and aging is urgently needed. Longevity is an important phenomenon in healthy aging. The present review summarizes the characteristics of longevity in the elderly population in Bama, China, where the proportion of centenarians is 5.7-fold greater than the international standard. We examined the impact of genetic and environmental factors on longevity from multiple perspectives. We proposed that the phenomenon of longevity in this region is of high value for future investigations in healthy aging and aging-related disease and may provide guidance for fostering the establishment and maintenance of a healthy aging society.

Virtually every country in the world faces enormous challenges as the global population ages. According to World Population Aging 2019 released by the United Nations and the current trajectory, the population aged over 80 years will triple to 426 million, the population aged 65 years or over will double to 1.5 billion, and one in six people will be older than 65 years by 2050. The health risks posed by aging can bring enormous burdens on families, health systems, and social protection and security. The incidences of age-related cerebro-vascular diseases, type 2 diabetes mellitus (T2DM), cancer, and neurodegenerative diseases are rapidly rising as the elderly population sharply rises [1-5]. As medical technology advances, most of the elderly choose to live longer in sound health. Healthy aging is a crucial component of anti-aging and longevity and has been the ultimate goal of this research field. Researchers have conducted a series of studies on longevity population cohorts from several countries, including China, the United States, Italy, and Japan [6-9]. They used the long-lived population as the research model, summarized their characteristics, explored the potential mechanisms of longevity, and endeavored to elucidate the healthy aging process. Bama, a multi-ethnic village in Guangxi, China, is very famous for the longevity of its local population. The proportion of people aged 100 years or over in Bama is 5.7-fold greater than the internationally defined longevity standard, according to the data of the Seventh Census in China (www.bama.gov.cn/sjfb/tjgb/t9295862.shtml). The longevity phenomena in Bama are intriguing as the region has a closed, unique natural environment and a stable genetic background. Here we review a series of relevant studies on Bama phenomenon, analyze the demographic data about long-lived population using geographic information system (GIS), and further discuss the multiple-dimensional factors including genetic factors, diet, natural environment, social factors, and so on, in order to obtain beneficial lessons from the longevity village of Bama and provide novel insights into healthy aging.

**Figure 1.**
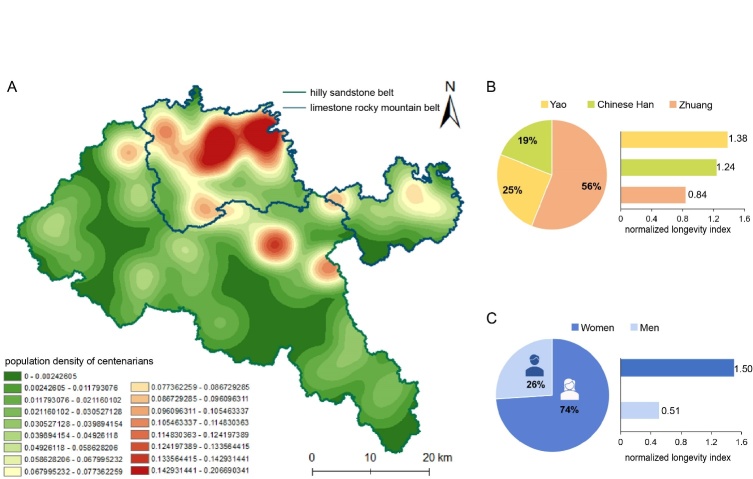
Distribution patterns of the centenarians in Bama, China. (A) Spatial distribution of the centenarians in Bama. Shown is a hotspot map by GIS. (B) Representative ethnic distribution of the centenarians in distinct ethnic groups of Bama. Left panel, proportion of the centenarians by ethnic group; right panel, data analysis of the normalized longevity index for centenarians. The index is defined as the ratio of the proportion of long-lived persons the indicated group to the proportion of this group in the total population. (C) Gender proportion in the centenarian population of Bama. Left panel, proportion of the centenarians by gender; Right panel, data analysis of the normalized longevity index for centenarians.

Recently, a public data survey (www.bama.gov.cn/sjfb/tjgb/t9295862.shtml) showed that the long-lived people of Bama have several characteristics in common, as shown in Figure 1. (1) The regional distribution of the long-lived elderly in Bama shows spatial aggregation by using GIS to sort out the data. Bama has typical karst landforms, and limestone rocky mountain and hilly sandstone belts comprise most of the landscape. The hilly sandstone and limestone rocky mountain belts account for ~69% and ~30% of the total karst landform in the area [10]. However, the long-lived population is distributed mainly in the limestone rocky mountain belt. Demographics show that there are approximately 56 persons over 90 years of age per 10,000 in the ten towns of the county situated in the limestone rocky mountain belt (Fig. 1A). About 12 of these individuals are centenarians. There are approximately 34 people over 90 years of age per 10,000 in the hilly sandstone belt and at least two of them are centenarians. (2) They are mostly from ethnic minorities. As of 2019, Bama had 12 ethnic groups including Yao, Zhuang, Han, Miao, Maonan, Mulao, Hui, Shui, and others. The minority population accounted for 84.68% of the total in the county and the Zhuang and Yao populations accounted for 66.30% and 18.08% of the minority population, respectively. The Zhuang and Yao minority accounted for 69% and 16% of the elderly over 90 years of age, respectively, while Chinese Han accounted 15%. Importantly, the Zhuang, Yao, and Han accounted for 56%, 25%, and 19% of the centenarians, respectively (Fig. 1B). Data analysis shows that Yao minority appears to have highest normalized longevity index of the centenarians among the different ethnic groups, implying a positive link between the genetic background of Yao and longevity in Bama. (3) There are gender differences in the distribution of the long-lived population in Bama. Of all centenarians in Bama, 74% are women and 26% are men; the normalized longevity index of women is 2.94 times than men, indicating that women may live longer than men in Bama (Fig. 1C). This finding is basically consistent with the results of prior studies on the relative proportions of men and women in the long-lived population of other regions [11-13]. (4) The long-lived population of Bama is characterized by familial aggregation. Sixty-five families have members who are over 90 years of age. The number of people surnamed Huang, Wei, and Luo are substantially more than that of others, among the 907 people over 90 years of age. The longevity phenomena of these three superfamilies deserves further investigation in the next step. (5) The longevity phenomenon in Bama is unique from an economic perspective. Actually, income has a complex impact on human life expectancy, while Bama is a rural area with a relatively low economic status. As a neighboring county with resembling GDP to that of Bama, Nandan County has a centenarian population density of 8/100,000, which is much lower than Bama, 43/100,000. Additionally, Bama has a relatively closed natural environment, low population mobility, relatively stable genetic background, and well-preserved ethnic culture; therefore, longevity characteristics that uniquely differ from other regions. In general, the elderly people of Bama have relatively long-life expectancy, good physical and mental health, and low prevalence of aging-related diseases [14, 15]. Hence, Bama is an ideal subject for research into healthy aging. We summarize the distribution patterns of the long-lived elderly in Bama, analyzed the potential factors and candidate factors that may affect longevity combining with the local characteristics of Bama, so as to shed light on the future research on longevity and aging, as well as proposal useful strategies promoting healthy aging [16].

**Figure 2.**
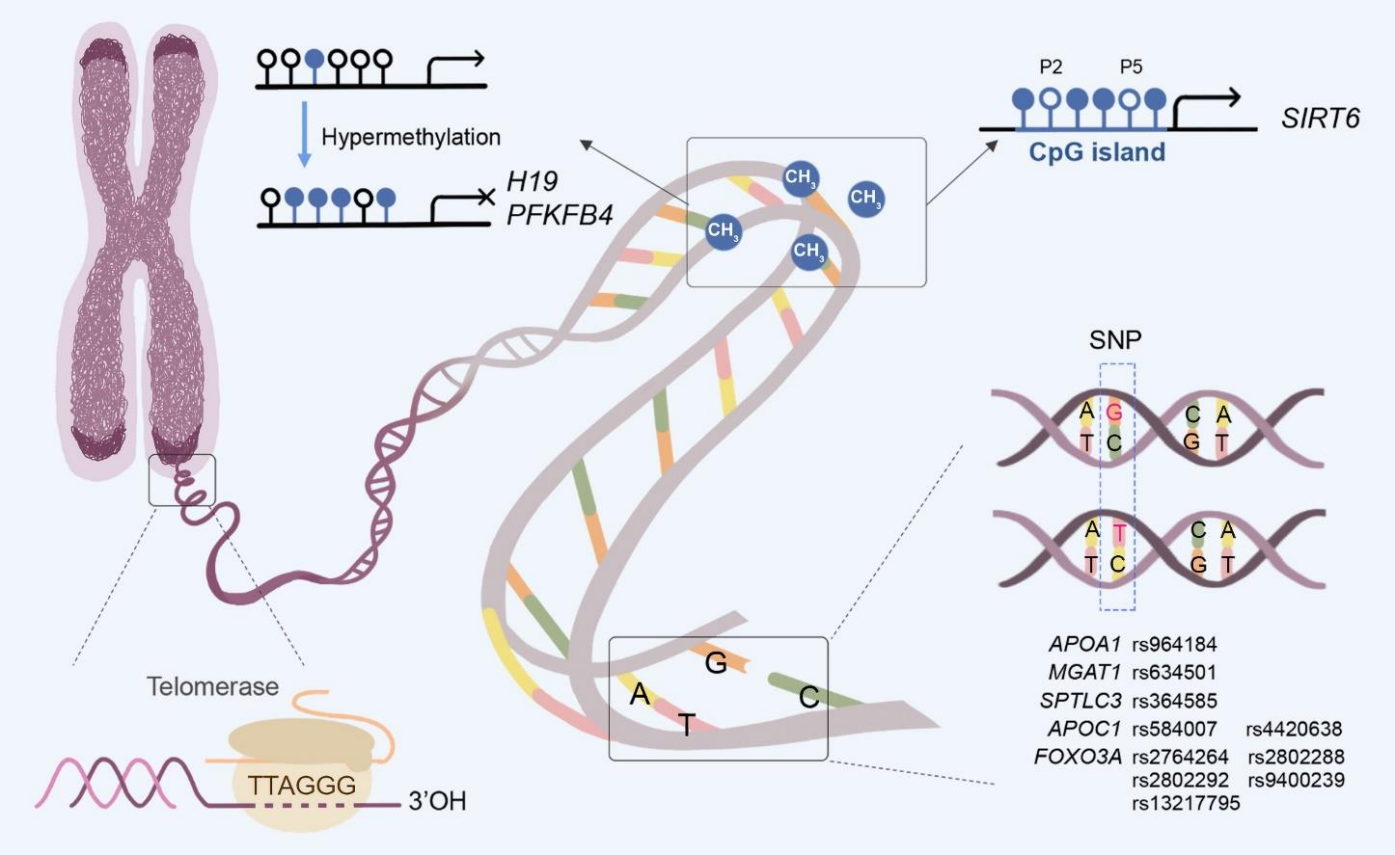
Main specific genetic factors are related to longevity in Bama, China.

## 1. Genetic factors

Longevity and aging are the consequences of interactions among multiple genetic and environmental factors [17]. Biologists empirically confirmed that lifespan and aging have genetic traits [18, 19]. Genetic traits account for 25% of all factors affecting lifespan [20, 21]. Hence, it is necessary to study lifespan from a genetic perspective. The people living in Bama are known for a family history of longevity. For three consecutive generations, there have been nonagenarians in Bama and the siblings of the elderly there also live long [22]. Bama has relatively stable heredity because of the local geography and ethnic customs. It is useful to dissect out the naturally occurring genetic variation and hereditary features in the long-lived population of Bama that will help to establish the roles of the specific genetic factors in regulating human lifespan. Actually, genetic research on longevity and aging has been conducted worldwide. In the present review, we are firstly focused on the relationship between Bama longevity and genetic factors such as gene polymorphism, DNA methylation, telomere length, and so on [23-27].

### 1.1 Gene polymorphism

Prior research on Bama focused on the impact of gene polymorphism and lifespan on intermediate cardiovascular disease phenotypes such as blood lipid levels. Several gene single nucleotide polymorphisms (SNPs) may be associated with lifespan and blood lipid parameters of the local population (Fig. 2). Such SNPs were identified by analyzing blood samples drawn from people in Bama at the core of the Hongshui River Basin [23, 24]. People with a family history of exceptional longevity have elevated high-density lipoprotein cholesterol (HDL-C) and low triglyceride (TG) levels [23]. These characteristics may reduce the risk of cardiovascular disease (CVD) in the elderly [28, 29], compared with those without exceptional longevity. The differences in serum HDL-C and TG levels between the two groups may be related to rs584007 and rs4420638 SNPs (Fig. 2) [23]. Consistently, the association between *APOC-I* rs4420638 and longevity was also reported on long-lived people in Europe [30]. The longevity-related gene *FOXO3A* has been found to exist eight SNPs, including rs2764264, rs9400239, rs13217795, rs2802288, rs2802292, rs28002290, rs7762395, and rs13220810, that are closely associated with human longevity [31, 32]. Five main haplotypes constructed from these SNPs were found in Bama population; two haplotypes of them, AG and TTTGTC, were found to be strongly linked to exceptional longevity phenomena of Bama [24]. Meanwhile, the minor allelic frequencies (MAFs) of rs2764264, rs9400239, rs13217795, rs2802288, and rs2802292 SNPs of exceptional longevity group were significantly higher than that of control group, showing that these five SNPs are related to exceptional longevity phenomena in Bama (Fig. 2) [24]. This finding was consistent with the observation in European longevity regions [31, 32].

Bama is a multi-ethnic region. Certain ethnic groups live in relative isolation, have unique lifestyles and diet habits, strict intra-ethnic marriage customs, and distinct ethnic origins and phenotypes. Certain lipid metabolism-related genetic characteristics and genotypes may differ between the ethnic minorities of China and the Han Chinese. Blood lipid profiles differ among ethnic groups [25-27]. About 40-60% of the variation in serum lipid profiles is genetically determined [33, 34]. The A allele carriers of the *MGAT1* SNP rs634501 in the Maonan ethnic group have higher APOB and lower APOA1 levels than other ethnicities [25]. The G allele carriers of the *APOA1* SNP rs964184 have lower HDL-C levels than other ethnicities [26]. The A allele carriers of the *SPTLC3* SNP rs364585 in the Jing ethnic group have higher TC and LDL-C and lower HDL-C levels than other ethnicities [27]. Thus, there may be racial/ethnic-specific associations between SNPs and serum lipid levels. The use of these minority populations facilitates the study of the genomic diversity and population genetics of longevity.

Currently, Apolipoprotein E (*APOE*) is also regarded as a common longevity-related variant gene that plays an important role in lipoprotein regulation [35]. Its role has been confirmed by the studies about Bama population [36, 37]. The heredity of the *APOE* genotypes is rather stable in the longevity families of Bama, as evidenced by the fact that there was no statistically significant difference in *APOE* genotypes frequencies between young and old subjects in the longevity family [38]. A recent gene-based association study of blood lipid levels in > 170,000 individuals with multiple ancestries and in different regions revealed rare coding variants in 35 genes including *CD36*, *CETP*, *SCARB1*, *LDLR*, and *APOC3* related to circulating lipid levels [39]. Variants of the gene encoding angiotensin-converting enzyme (ACE) might also be associated with longevity. A meta-analysis of Caucasians, Chinese, and Koreans disclosed that ACE D alleles and DD genotypes moderately but significantly increase longevity [40]. Certain metabolism- and immune system-related genes are also associated with longevity. A genotype of *IGF-1* related to low serum protein levels is overexpressed in long-lived people [41]. A non-synonymous mutation in *IGF1R* is comparatively more common in female centenarians [42]. The involvement of the immune-related *IL-6* and *TGF* in genetic human lifespan variations was demonstrated in a study on an Italian population [43]. Investigations of the associations among genetic polymorphisms and longevity have been performed globally. As discussed above, emerging evidence suggests that the gene polymorphisms are related in part to longevity in Bama, since a few of gene SNPs have been detected in several studies [23-27]. Further functional studies need to be conducted in the next step that will greatly help to clarify the relationship between gene polymorphism and longevity.

### 1.2 DNA methylation

Recent studies suggested that DNA methylation is related to age-related sites and longevity [44-46]. In the same area of Bama, the long-lived family had 117 hypermethylated genes compared with non-the control family [47]. A gene annotation analysis showed that these hypermethylated longevity-related genes were enriched in the chemokine signaling and natural killer (NK) cell-mediated cytotoxicity pathways of cellular immunity [47]. Cytotoxic T lymphocytes and NK cells were also detected in the Japanese centenarians with superior resistance to various diseases [48]. Most centenarians maintained good states of lymphocyte proliferation, phagocytosis, and NK cells activity which extended their five-year lifespans [49, 50].

DNA methylation is an epigenetic modification that plays an important role in regulating gene expression [51]. Genes associated with disease occurrence may also be epigenetically modified and implicated in the health promotion and longevity phenotypes [52]. According to the “hypermethylation suppressing gene expression” theory [53, 54], *H19* and fructose-6-phospho-2-kinase/fructose-2,6-bisphosphatase (*PFKFB4*) were downregulated in the people of Bama with a family history of longevity (Fig. 2) [47]. *H19* is a disease susceptibility gene that is upregulated in various cancers [55, 56]. *H19* methylation and inhibition may help people avoid lethal age-related diseases, thereby increasing the possibility of living longer [47, 57, 58]. *PFKFB4* is a key regulatory enzyme of glucose metabolism and the main energy source of various malignant tumor cells [59]. *PFKFB4* downregulation inhibits tumor cells through glucose restriction [60, 61]. *PFKFB4* and *H19* are in a similar state in people with a family history of longevity. *PFKFB4* downregulation is a protective factor for the people in Bama as it may protect them from age-dependent malignancies [47]. In contrast, the rates of *SIRT6* methylation were significantly decreased at the P2 and P5 CpG in the Bama longevity population. Hence, they had elevated *SIRT6* mRNA levels. The level of SIRT6 protein is negatively correlated with methylation rate at the P2 and P5 CpG sites (Fig. 2) [62]. SIRT6, a member of the SIRT family, usually functions as a protein deacylase [63]. It regulates DNA repair, stabilizes the epigenome [64] and, by extension, prevents age-related diseases and extends a healthy lifespan [65]. Low methylation levels of SIRT6 lead to high SIRT6 protein expression. The latter may be a protective factor in the elderly population of Bama. Previous study has sequenced the genome-wide methylation profiles of four Chinese centenarians and four middle-aged controls [66]. They identified 626 regions significantly differing in terms of methylation. A subsequent analysis revealed that the genes associated with these differential methylation regions (DMRs) were significantly enriched in aging-related diseases including CVD, T2DM, stroke, and Alzheimer's disease. A comparison and an analysis of the genome-wide methylation data for Caucasian centenarians validated these discoveries. Previous investigations sought longevity-related genomic variations. The aforementioned studies consistently demonstrated that the elderly had a unique DNA methylation pattern that delays the onset of aging-related diseases and favors longevity by repressing the genes associated with susceptibility to them. DNA methylation and its variations at specific CpG islands were conserved in the offspring of the centenarians [62, 67, 68].

DNA methylation is usually in dynamic equilibrium. As the methylome operates at the interface between the genome and the environment [69], the methylation level may change in response to exercise [70, 71], diet [72], smoking [73, 74], and pollutants [75]. Aging-related diseases caused by epigenetic alterations such as DNA methylation are prevented and controlled by inhibiting or promoting their associated regulatory enzymes [76]. There is growing research interest in the development and administration of epigenetic medicines and diets [76]. Interventions targeting epigenetic information could potentially extend lifespans, counteract aging-related diseases, and favor healthy aging [19].

### 1.3 Telomere length

Genetic and environmental factors affect telomere length [77]. Average telomere length decreases with age because the telomere tandem repeat "TTAGGG" is lost during cell division (Fig. 2) [78]. Telomere shortening can trigger a number of secondary pro-aging phenomena such as increased DNA damage and genomic instability, cellular senescence and/or apoptosis, *etc.* [79]. Thus, it is always considered as an indicator for cellular senescence [80-82]. Telomere length was negatively correlated with aging in the Bama population [83]. Long-lived individuals and their offspring had longer telomeres than the general population [84]. In Bama, 70% of the telomere length in is heritable according to a clear maternal and paternal genetic model spanning > 2 generations [83]. Telomerase may preserve telomere length, repair telomeres, and synthesize telomere DNA [85]. Telomeres and telomerase play universal roles in aging characteristics, progeria, and aging-related neurodegenerative diseases and cancers [86]. In Bama, however, the relationships among telomere, telomerase, and longevity are unclear. An earlier study showed that the telomere lengths of German Jewish centenarians and their descendants were longer than those of the control possibly because of synonymous and intron mutations of the telomerase reverse transcriptase gene and telomerase RNA [87]. Another study on the long-lived people of Costa Rica shows that dietary factors and particularly traditional food patterns were related to telomere length and might have helped to prolong the lifespans of the elderly there [88].

Current research on telomeres and aging indicates that telomere dysfunction attenuates mitochondrial biogenesis and function, downregulates genes encoding antioxidant defense, and accelerates aging [89]. Epigenetically, telomeres control aging by regulating SIRT which, in turn, affects metabolism, antioxidant defense, and stress tolerance [90, 91]. Inflammatorily, telomere dysfunction may also stimulate proinflammatory factor production and secretion [92] and generate extrachromosomal fragments that promote autophagic cell death [93]. The studies of telomere length on Bama longevity are shallow, just measure the telomeres length of long-lived people and their offspring, but the specific mechanism is still unclear. Some literatures have suggested that change in leukocyte telomere length may be a more relevant metric than static levels [94, 95]. Hence, conducting in-depth investigations on telomeres and human longevity using the longevity resources of Bama combined with various methods to measure telomere length [96, 97] may provide strategies for the treatment of aging and aging-related diseases and the prognostication of advanced cancers.

## 2. Dietary patterns

**Figure 3.**
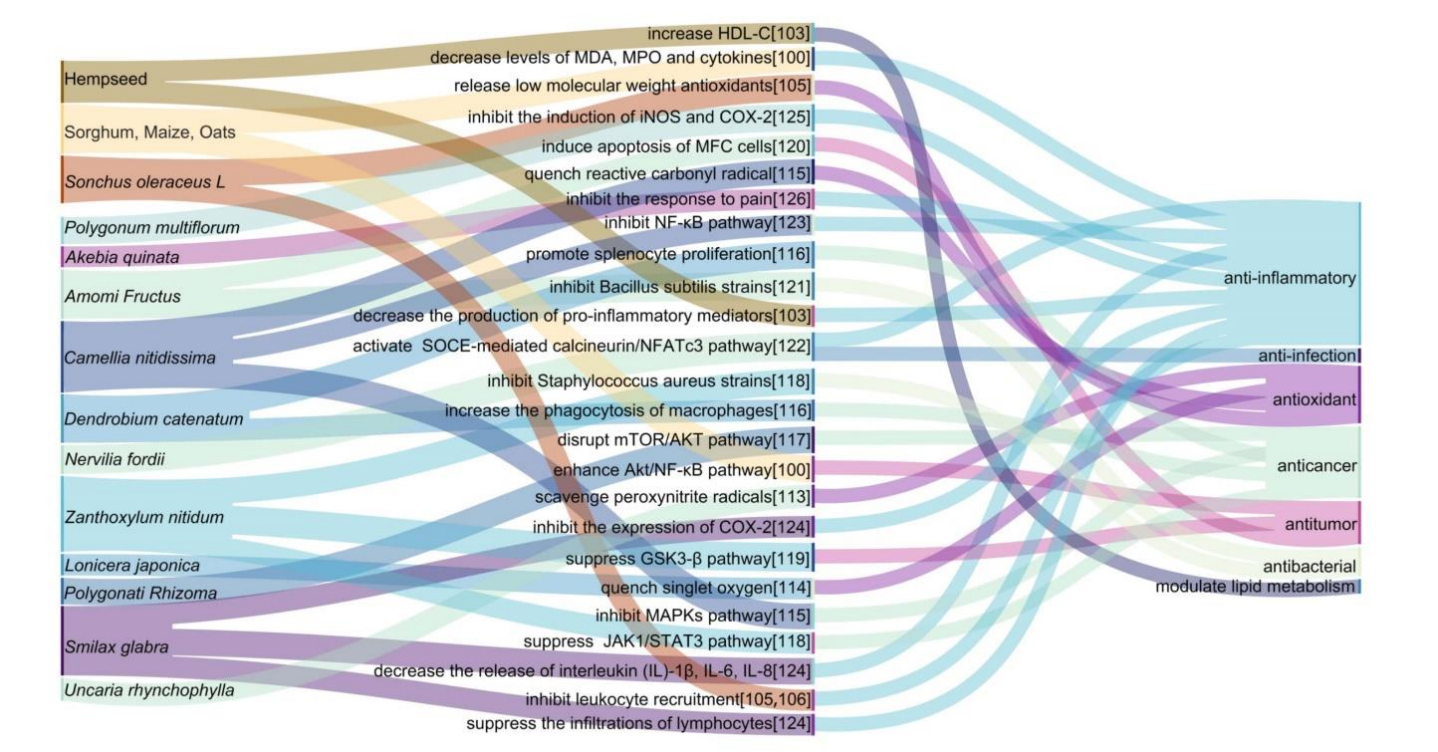
Sankey diagram of the function of special foods and medicinal herbs in Bama, China.

The influence of diet on lifespan is complicated, thus the effects of any single nutrient or food on health should not be overstated [98]. By contrast, the health impact of multiple foods and nutrients in a complete diet merits serious consideration. The long-lived population in Bama consumed a fiber- and polysaccharide-rich diet with "five low, two high" characteristics, namely, low fat, animal protein, calorie, salt, and sugar content and high vitamin and fiber intake [99]. The basic diet in Bama consists of porridge, coarse grains, dark vegetables, and livestock meat (Fig. 3) [99, 100]. In contrast, the characteristic of dietary patterns in developed areas is typically "high oil and high salt". Specialty foods in Bama include hemp (*Cannabis sativa* L.) seeds, sow thistle (*Sonchus oleraceus* L.) (Fig. 3) [101], and Bama miniature pig. The residents of Bama use hemp seeds to make edible cooking oil [99]. Hemp seeds grow naturally in the limestone rocky mountain area of Bama and the hemp plants are free of pesticides and fertilizers. Hemp seeds contain the proper ω-6:ω-3 fatty acid (FA) ratio which can increase serum HDL-C, regulate lipid metabolism, and inhibit the production of proinflammatory cytokines [102, 103]. Bitter vegetables such as *Sonchus oleraceus* L. are widely distributed in Bama. *Sonchus oleraceus* L. contains abundant antioxidants, ω-3 FA, phenols, and flavonoids [104]. It protects cells from stress-induced aging, inhibits leukocyte recruitment, and has anti-inflammatory efficacy [105, 106]. *Sonchus oleraceus* L. has potent antioxidant activity as it releases low-MW antioxidants [107]. Diet also regulates gut microbiota community structure and function [108]. The foregoing diet composed of hemp seeds and bitter vegetables reduced the abundance of lipopolysaccharide (LPS)-producing *E. coli* and increased the abundance of beneficial *Bifidobacterium* and *Lactobacillus* in mice [101]. This diet promotes health and longevity, which has also been verified in mice [101].

The Mediterranean diet, a widely recommended traditional healthy diet, is also associated with longevity and vitality as it is characterized by high fiber and vitamin intake, low fat intake, and a high ratio of monounsaturated to saturated FA [109]. The Mediterranean diet consists of olive oil, vegetables, fruits, beans, nuts, seeds, fish, and seafood [98]. This dietary pattern resembles that of the Bama diet. The latter emphasizes the intake of vegetables, grains, and vegetable oil but only small quantities of meat. The Mediterranean diet maintains cognitive ability, reduces the risks of CVD and cancer [110, 111], and is strongly associated with lowering mortality in people over 65 years of age [112]. Hence, the effects of a mixed diet rather than single food items or nutrients on health and longevity should be investigated. Future research should explore the correlation between dietary patterns and the incidence rate of age-related diseases in Bama. However, it is difficult and even impractical to separate the influences of diet from complex factors such as lifestyle and cultural customs. Current scientific research on the Bama diet is not systematic. As the "five low, two high" standard of the Bama diet has neither been quantified nor unified, research on its effects on aging and longevity is restricted. Animal models could also be used in the future to segregate the influences of dietary factors from those of lifestyle and cultural customs on longevity and aging in Bama.

## 3. Medicinal herbs in Bama

The natural environment of Bama has high biodiversity and abundant plant resources with potential medicinal value. There are approximately 562 different medicinal plants in Bama including *Lonicera japonica*, *Zanthoxylum nitidum*, *Akebia quinata*, *Polygonati Rhizoma*, *Smilax glabra*, *Polygonum multiflorum*, *Uncaria rhynchophylla*, *Amomi Fructus*, *Nervilia fordii*, *spatholobus Suberectus Dunn*, *Dendrobium catenatum*, and *Camellia nitidissima* (www.bama.gov.cn/zjbm/zrdl/t6215241.shtml). Many researchers have extracted a large number of natural compounds from these medicinal herbs to explore their effects on diseases. These natural compounds exhibit antioxidant [113-115], anticancer [115-119], antitumor [118, 120], antibacterial [118, 121], anti-inflammatory [122-128], or anti-infection [122] efficacy that may be involved in longevity (Fig. 3). For example, nitidine chloride from *Zanthoxylum nitidum* can reduce inflammation by inhibiting the NF-κB pathway, as well as inhibit tumorgenesis by inactivating the JAK1/STAT3 pathway [118, 119]. Flavonoids in *Camellia nitidissima, Smilax glabra*, and *Nervilia fordii*, were found to prevent inflammation and carcinogenesis by inhibiting the formation of glycation end-products, inactivating the NF-κB and MAPK pathway, and preventing the release of IL-1 and IL-6 [115, 123, 124]. The rhamnocitrin in *Nervilia fordii* can inhibit the activation of the SOCE-mediated calcineurin/NFATc3 signaling pathway, suppress vascular endothelial activation, and thus may exert anti-inflammatory effects [122]. In fact, people in Bama always prefer to self-treat common illnesses by taking medicine herbs in such an area with limited access to modern medical resources. The medicinal value of herbs needs to be further explored by more studies in the future, providing novel insights into the regulation of lifespan.

## 4. The natural environment

Environmental factors have an enormous impact on human life expectancy and even alter genetic traits [129]. The correlation between longevity and environment is a hot research topic in modern gerontology, as this association may reflect the proclivity of healthy aging. Bama is in the subtropical zone of China and is characterized by moderate temperatures and abundant rainfall. The woodland has dense plant populations, the residential areas are scattered, and there is only sparse cultivated land. The original natural environment is well preserved and seldom developed and has good air quality. Various unique ecological elements contribute to the harmonious natural habitat in the region. The residents of Bama enjoy leisurely, peaceful lives with less mental anxiety and stress. Thus, the social environment there is calm and serene. The water and soil of Bama abound in trace elements [130], the geomagnetic field has an appropriate "power window" [131], and the sunshine duration is appropriate [132]. These factors affect the quality of human life and may be correlated with the longevity phenomenon observed in Bama. Relevant research on the elderly population in this region will help elucidate the complex influences of the environment on lifespan. These learnings may facilitate the development and implementation of measures preventing unhealthy aging, establish a suitable living environment, and promote a healthful lifestyle.

### 4.1 Trace elements

Bama is located in the subtropical zone of China and has typical karst landforms [10]. The stratum consists mainly of limestone and carbonate and has a high mineral content [130, 131]. The soil is rich in trace elements such as Ca, Co, Cr, Mg, Mn, Na, I, Fe, and K [130]. The trace elements in soil affect water quality, the atmosphere, and plant, animal, and human health through the food chain [133-136]. Though trace elements comprise only a minuscule portion of the human body, they nonetheless have a significant impact on it. Trace element toxicity and deficiency cause specific and, in certain cases, serious disorders. Magnesium inhibits certain enzymes in lipid metabolism and regulates the function of *N*-methyl-*D*-aspartic acid (NMDA) receptors [137, 138]. Thus, Mg helps prevent certain aging-related conditions such as Alzheimer's disease, insulin resistance, T2DM, hypertension, and CVD [139]. A recent study suggested that high Mg intake (39 mg/mL Mg^2+^ 15 g/L MgCl_2_) delayed aging in a premature aging model mouse [140]. The soil Cd, Co, Mg and drinking water Co concentrations were significantly higher and the soil and drinking water Na and hair Mg, Na, and Mn concentrations were significantly lower in the longevity than the non-longevity areas in Bama, within a normal range. Adequate soil Mg may be conducive to long lifespans while excess soil and drinking water Na and hair Mg, Mn, and Na may shorten lifespans [130]. Bama has abundant manganese ore. However, Mn ore mining and processing might cause manganese pollution in the air and water, and factory workers and people living adjacent to the mines and smelters may be exposed to excessively high Mn levels. Mn regulates glucose and lipid metabolism, accelerates protein and vitamin synthesis, controls the endocrine system, and maintains normal immune function [141]. Nevertheless, excess Mn crosses the blood-brain barrier (BBB), damages the central nervous system (CNS), and causes learning and memory disorders [142] and Parkinson-like symptoms [143]. Essential trace elements work synergistically in the human body and their levels were in proper balance in the long-lived population. Long-lived people may have a unique "longevity element spectrum" characterized by a healthy balance between the synergistic and antagonistic efficacy of trace elements.

### 4.2 Geomagnetic intensity

Research in geomagnetic biology evaluates whether human life is affected by the magnetic field of the Earth. Bama is located in a fault zone and has a limestone rocky mountain area on one side and a hilly sandstone area on the other [130, 131]. The limestone rocky mountain area is rich in Mn, Zn, Mg, Fe, and other minerals that are magnetized under the influences of the geomagnetic field. Hence, the geomagnetic intensities of the villages in limestone rocky mountain areas are generally higher than those of the villages in hilly sandstone areas. This pattern is roughly consistent with the spatial distribution of the long-lived population in Bama [131]. A "power window" curve describes the relationship between geomagnetic field intensity and longevity in Bama villages [131]. Nevertheless, few studies to date have investigated this association in Bama. An appropriate geomagnetic field promotes cell growth [144], activates cells [145], balances endocrine disorders [146], promotes blood circulation [144, 147], promotes inflammation regression [148], reduces swelling and pain [149], and regulates blood pressure [150]. However, there is still insufficient evidence to indicate that the predicted geomagnetic density has greater therapeutic efficacy than others. The complexity of the biological effects of the magnetic field makes research on its association with human health challenging. In the future, further analysis of the correlation between the geomagnetic field and the physiological state of Bama population may provide meaningful clues for the impact of magnetic field on human health and aging.

### 4.3 Sunshine duration

The region of Bama also has a unique sunshine pattern. The long-lived people of Bama tend to congregate in the karst regions of the northern and southwestern parts of the county. These areas have highly variable topography and unevenly distributed sunshine resources. Terrain shading and atmospheric cloud occlusion significantly affect the spatial distribution of sunshine duration, which is characterized by a unique, regular, non-latitudinal pattern. There is a clear seasonal imbalance in the daily real illumination hours in Bama County. The highest values occur in summer (May to September) while the lowest occur in winter (December to February) [132]. Previous research showed that the longevity of the adult citrus whitefly (*Dialeurodes citri*) decreased with increased sunlight exposure duration [151]. Excessive sunlight exposure increases the risks of skin aging and cancer in humans [152]. The Far-infrared Ray (FIR) generated by appropriate solar radiation increases arterial blood flow, improves peripheral blood circulation and endothelial function, lowers blood pressure, and promotes capillary dilatation [153]. Thus, appropriate sunlight duration may be conducive to human health. The characteristics of sunshine duration in Bama are partially consistent with the geographic distributions of the long-lived people there. Nevertheless, the precise correlations between these factors and longevity remain to be empirically determined.

## 5. Summary and Perspectives

In general, the factors that influence longevity and lifespan are related to heredity (nature) or environment (nurture) [17, 154]. Here, these above studies conducted on the long-lived population in Bama, China, have given us some important lessons: (1) Several SNPs elevate HDL-C and lower LDL-C and TG levels, which may reduce the risk of cardiovascular diseases; (2) DNA methylation may repress the expression of susceptibility genes and increase that of longevity genes; (3) Telomere length is heritable and may affect antioxidant efficacy, tolerance and inflammation, respectively; (4) The diet pattern consisting of hemp seeds and *Sonchus oleraceus* L. *etc*. of Bama is characterized by high fiber, vitamin, but low animal fat and protein, sugar, salt, calorie; (5) Abundant natural compounds extracted from medicinal herbs in Bama, which probably play regulatory roles in anti-oxidation, anti-cancer, anti-inflammatory or other biological effects that may relate to healthy aging. (6) Some beneficial natural environmental factors, including trace elements, geomagnetic intensity, sunshine duration, *etc.*, seem to contribute to the formation of Bama longevity phenomena to some extent.

Of note, the population density of centenarians per 100,000 people in Bama was twice as high as it was in Shanghai according to 2020 census data, despite the socioeconomic level of Bama is far lower and its medical resources is fewer than that of Shanghai. In fact, combining sociological and scientific methods have been used to reduce the error of age statistics in many longevity studies of Bama, including evaluating the bone age, checking registered residence, genealogy, and investigating social relations [23, 155]. This interesting phenomenon indicates that the socioeconomic factor is not the primary determinant among many factors affecting longevity population in Bama. The low economic level is often accompanied by the lack of education [156]. According to the demographics, more than half of long-lived elderly of Bama have no schooling (www.bama.gov.cn/sjfb/tjgb/t9295862.shtml). Thus, it's hard to discuss the direct relationship between school education and longevity in Bama. However, humanistic education seems to be more beneficial from their unique ethnic culture that shapes their belief, thinking, and lifestyle [156, 157], with sustainable trans-generation preservation. Since ancient times, people here believe that benevolent people live longer and thus have little mental stress with a simple lifestyle. Additionally, regular labor has been integrated into their daily life. Taken together, we suggest that more potential factors should be investigated from the Zhuang & Yao culture in future studies on longevity.

The factors affecting longevity are rather complex [158], and it is limited to analyze the phenomenon of longevity only from a single perspective. The probable involvement of numerous and complex factors with little effects are features that will produce many chance findings. In Bama, insufficient long-term follow-up of the population and the limited sample size of the survey population are common problems with the longevity study of Bama as well as other longevity studies worldwide [159-161]. Besides, in studies on longevity, observational research often emerges [162-164], but has disadvantages of subjective judgement [165]. Thus, further research requires more strict screening population mechanism [35]. It is used to collect more longitudinal data accompanied by extensive phenotyping, which would be required to evaluate the joint impact of multiple factors on lifespan and shift the focus from longevity to a better understanding of healthy aging [16, 166, 167].

Future investigations are necessary to establish genetic resource samples, biological, and environmental specimen banks for the long-lived population cohort in Bama to provide standardized biological materials and data for longevity and aging-related research. Go a step further, investigations should endeavor to build and implement a health record sharing and stream processing platform, long-term population queues, and comprehensive analytical systems generating big data and interaction mechanisms. On this basis, the genetic characteristics of longevity in Bama will provide more evidence for the wide genetic heterogeneity characteristic of humans. The latter is the basis of precision medicine [168]. This discipline aims to identify the key genetic determinants of disease and customize interventions and treatments based on these unique genetic variations. Personalized treatment based on unique gene mutation research and multi-target drug development based on medicinal herbs provide new directions for the advancement of gerontology [169]. Using some candidate longevity genes screened from Bama longevity population to explore these genes and the molecular mechanisms of aging can provide novel insights into aging interventions. Additionally, the living environment and habits of the elderly in Bama should be closely examined and evaluated as they are critical indices of the health status of this population and strongly influence the results of the evaluation. Tracking the long-lived population of Bama would also provide a useful tool for related research in other regions, as well as facilitate the design and implementation of a new health management model and a system of real-time, dynamic population data collection. In-depth research into the living environment and lifestyle of the long-lived elderly should aim towards defining a healthy, proactive lifestyle and providing a scientific reference enabling the urban elderly to achieve the goal of healthy aging. The longevity studies in Bama indicate that formulating differentiated population aging strategies may guide the establishment and maintenance of a healthy aging society.
